# Evaluating Biomarkers in Melanoma

**DOI:** 10.3389/fonc.2014.00383

**Published:** 2015-01-23

**Authors:** Panagiotis Karagiannis, Matthew Fittall, Sophia N. Karagiannis

**Affiliations:** ^1^St. John’s Institute of Dermatology, Division of Genetics and Molecular Medicine, King’s College London, London, UK; ^2^NIHR Biomedical Research Centre, Guy’s and St. Thomas’ Hospital, King’s College London, Guy’s Hospital, London, UK; ^3^Clinical Oncology, Guy’s and St. Thomas’s NHS Foundation Trust, London, UK

**Keywords:** biomarkers, melanoma, humoral immunity, antibodies, cancer, prognosis, immune response, inflammation

## Abstract

The incidence of cutaneous melanoma has more than doubled over the last decades making it one of the fastest rising cancers worldwide. Improved awareness and early detection of malignant moles now permit earlier diagnosis aiming to decrease the likelihood of recurrence. However, it is difficult to identify those patients initially diagnosed with localized melanoma who subsequently develop metastatic disease. For this group, prognosis remains poor and clinical outcomes are variable and challenging to predict. Considerable efforts have focused on the search for novel prognostic tools, with numerous markers evaluated in the circulation and in tumor lesions. The most reliable predictors of patient outcome are the clinical and histological features of the primary tumor such as Breslow thickness, ulceration status, and mitotic rate. Elevated serum levels of the enzyme lactate dehydrogenase, likely to indicate active metastatic disease, are also routinely used to monitor patients. The emergence of novel immune and checkpoint antibody treatments for melanoma and increasing appreciation of key roles of the immune system in promoting or halting cancer progression have focused attention to immunological biomarkers. Validation of the most promising of these may have clinical applications in assisting prognosis, assessing endpoints in therapy, and monitoring responses during treatment.

## Introduction

The incidence rates of melanoma are rising constantly, faster than for any other malignancy over the last two decades (1). Currently, melanoma has an incidence rate of 12.4/100,000 and a mortality rate of 3/100,000 in the UK (worldwide ASR 3.1/10000 and 0.8/10000, respectively) (2). Higher reported incidence rates partly reflect better surveillance and early diagnosis programs. Improvements in early detection could help identify patients at earlier, more curable disease stages, which may translate to enhanced overall survival (OS) rates (3). Currently, however, the number of patients dying of the disease is significantly greater than 1.3% diagnosed with incurable metastatic disease at presentation, implying a significant rate of disease progression in patients originally diagnosed with local disease (4–6). Treatment options for these patients are limited despite recently approved and emerging molecular targeted and immune therapies (7–9).

### Classification and staging

Cutaneous melanoma is classified into four types: (1) lentigomaligna melanomas with a papular or nodular structure; (2) superficial spreading (malignant) melanomas with large flat irregular pigmented lesions that grow laterally before invading the dermis; (3) nodular (malignant) melanomas with rapidly growing nodules that tend to ulcerate and bleed; (4) acral lentigo (malignant) melanomas mainly present at sites of friction, such as the sole, palm, or under nails.

Similarly, staging of melanoma is determined according to defined criteria (T: local extent of tumor; N: regional lymph-node involvement; M: distance metastasis-classification, Table 1). These parameters include the extent of dermal invasion (Breslow thickness and Clark’s level); the presence of ulceration; the mitotic rate of melanoma cells; and the presence of loco-regional or distant metastases. Although more advanced disease stage correlates with a worse prognosis, there have been reported cases of spontaneous melanoma lesion regressions and remissions of systemic disease, and this is perhaps attributed to immunological responses in these patients (4).

**Table 1:** **TNM classification**.

Stage	Primary tumor	Lymph node	Metastases
IA	<1 mm, no ulceration, mitoses <1/mm^2^ (T1a)		
IB	<1 mm with ulceration or mitoses ≥1/mm^2^ (T1b)		
	1.01–2 mm, no ulceration (T2a)		
IIA	1.01–2 mm, ulceration (T2b)		
	2.01–4 mm, no ulceration (T3a)		
IIB	2.01–4 mm, ulceration (T3b)		
	>4 mm, no ulceration (T4a)		
IIC	>4 mm, ulceration (T4b)		
IIIA	Any thickness, no ulceration (T1–4a)	1 nodal micrometastases (N1a); 2–3 nodal micrometastases (N2a)	
IIIB	Any thickness, no ulceration (T1–4a)	1 nodal macrometastases (N1b); 2–3 nodal macrometastases (N2b)	
	Any thickness, ulceration (T1–4b)	1 nodal micrometastases (N1a); 2–3 nodal micrometastases (N2a)	
	Any thickness, no ulceration (T1–4a)	In transit metastases/satellites, no metastatic nodes (N2c)	
IIIC	T1–4b	N1b–N2c	
	Any T stage	4 + metastatic nodes, or matted nodes, or in transit metastases/satellites with metastatic nodes (N3)	
IV	Any T stage	Any N stage	Distant skin, subcutaneous, or nodal metastases (M1a)
	Any T stage	Any N stage	Lung metastases (M1b)
	Any T stage	Any N stage	All other metastases or M1a/b sites with raised LDH (M1c)

### Loco-regional management

Localized disease in melanoma is currently treated by surgical excision with a 0.5 cm surrounding margin for tumor *in situ*, 1 cm margin for invasive melanoma of <2 mm Breslow thickness, and 2 cm margin for melanomas of ≥2 mm thickness (10, 11). Routine elective loco-regional lymphadenectomy has not been proven to be superior to observation alone (12). However, sentinel lymph-node biopsy has been suggested by several groups to be of benefit for patients with stage IB disease or higher, allowing for more accurate staging and therefore clearer information on prognosis (12). Radiotherapy could be used for primary lesions only if unresectable and for dissected lymph-node basins in patients at high risk of recurrence (13, 14).

### Systemic therapy

#### Conventional cytotoxic chemotherapy

Conventional cytotoxic drugs are largely ineffective in melanoma. Dacarbazine (DTIC), until 2011 the only standard therapy, has a low objective response rate (<20%) in metastatic disease (7). Numerous other chemotherapeutic agents including temozolomide, taxanes, fotemustine, and platin derivatives have been unsuccessfully trialed as single agents, combinations, or in conjunction with immune-modulators such as IFNα-2b or IL-2 at the cost of significant toxicity (14, 15).

#### Molecular targeted therapies

An activating mutation in the BRAF^V600^ gene on the long arm of chromosome 7 is found in approximately 50% of melanomas (16, 17). This change results in the constitutive activation of BRAF kinase, likely to promote RAS–RAF–MEK–ERK pathway-induced melanoma cell survival and proliferation. Vemurafinib is a pathway inhibitor drug that inhibits the mutated form of the BRAF kinase. Although originally designed to block the V600E (glutamate for valine) mutated form of BRAF, it can also inhibit other mutant forms such as the V600K (lysine for valine) (7, 18–20). In stage IV disease, vemurafenib has been shown to prolong progression-free survival (12.5 vs. 9.5 months) and OS (13.6 vs. 9.7 months) in comparison to dacarbazine (DTIC) (21). Vemurafenib, the first pathway inhibitor therapy approved for melanoma in 2011, represented the first significant change in outlook for stage IV disease in decades. However, following treatment, the majority of treated patients develop secondary resistance to the drug, thought to be associated with mechanisms overcoming the blockade of BRAF kinase inhibitors and with alternative activation of the MAPK kinase pathway (22). Treatment has also been linked to an enhanced risk of keratoacanthomas, squamous cell carcinomas (SCC), and the development of new melanomas (23–25) implying manipulation of other signaling cascades. Clinical trials are ongoing for BRAF inhibitors in the adjuvant setting and for combination therapies such as with BRAF and MEK inhibitor drugs. Recently, two trials reported that the combination of a BRAF inhibitor and a MEK inhibitor significantly improve progression-free survival (26, 27). Standard clinical evaluation now includes screening for the presence of the BRAF mutation in excised high risk and metastatic melanoma deposits. This test, now used for selecting patients to receive pathway inhibitor treatment, may be considered as a co-diagnostic tool for this treatment.

The identification of new mutations such as alternative BRAF, NRAS, c-kit, GNA11, and GNAQ mutations has led to the development of further kinase inhibitors and a number of these are being assessed pre-clinically and in clinical trials.

#### Immune therapies

The efficacy of immune therapies such as pegylated IFNα-2b and high dose IFNα-2b has been known for a number of years. These adjuvant treatments have shown improved disease-free survival (DFS), relapse-free survival (RFS) but only marginal improvements for OS (14, 28, 29). Their cost and toxicity, however, mean that their routine use is extremely limited. Likewise, high dose IL-2 was, until recently, the only effective therapy in stage IV disease, capable in rare cases of inducing prolonged remission (15). High toxicity confined treatment to a small subset of selected patients and in specialist clinical centers.

Ipilimumab is an IgG1 monoclonal antibody that recognizes the immune checkpoint cell surface molecule CTLA-4 on T cells. The antibody blocks the binding of the immunomodulatory molecule CTLA-4 to CD80/CD86 on the surface of antigen-presenting cells, preventing negative CTLA-4-mediated signals. This can result in universal activation of T cells, including some capable of specifically recognizing melanoma cells. Ipilimumab has shown efficacy in several clinical trials in untreated and treated metastatic patients in combination with a range of therapies such as peptide vaccines and as a mono-therapy (8, 30, 31). Some long-term responses are observed, albeit in a minority of patients and use of this agent is associated with autoimmune-like toxicities, most likely due to universal activation of CTLA-4-expressing T cells. The agent was approved by regulatory authorities in metastatic melanoma in 2011.

Based on the promise demonstrated by anti-CTLA-4 treatment, antibodies against other immune regulatory proteins such as PD-1, PD-L1, CD137, OX40, and CD40 are being investigated (32, 33). Administration of the anti-PD-1 antibody nivolumab demonstrated impressive objective responses in approximately 30% of individuals treated in a phase I trial (34). Combination therapy with nivolumab and ipilimumab in a dosing study demonstrated clinical safety and showed enhanced efficacy that appeared to be superior compared to data published on either antibody alone (35). Hamid et al. reported that the monoclonal antibody lambrolizumab (anti-PD-1) can induce sustained tumor regression in patients that had refractory disease despite ipilimumab treatment (36). Based on early clinical trial data, the anti-PD-1 antibody pembrolizumab was approved for the treatment of advanced melanoma in 2014 (37, 38).

The emergence of these new targeted and immune therapies, their success in only subsets of patient groups, as well as the need to assess patient benefit with combination treatments, all highlight the need to develop a range of tools, not only for prognosis but also to assist prediction and monitoring of treatment responses (39).

## The Search for Biomarkers in Melanoma

The rapidly evolving clinical landscape of melanoma management and therapy mandates the search for new candidate biomarkers. Clinical trials traditionally rely on broad clinical groups and long-term objective outcomes, for example, assessment of OS in patients with previously treated stage IV melanoma. Patient stratification is partly served by co-diagnostic tools, such as in the case of the BRAF mutation test, which can help stratify patients who may receive pathway inhibitor drugs; however, these co-diagnostics do not always provide prognostic or predictive information. Biomarkers could revolutionize the process of drug development and those predictive of response could rationalize entry to trials for those patients most likely to benefit (40). New reliable biomarkers are thus required for all stages of melanoma management to assist with early detection, diagnosis, staging, prognosis as well as prediction, and monitoring of treatment response.

Disease-relevant candidate markers for melanoma have traditionally been derived from dissecting melanoma disease pathways. A number of candidates have been studied in sera and tumor specimens (Table 2) and some of these are discussed here. For this, a literature search using the search engine “Pubmed” was conducted with the following keywords and phrases: melanoma, malignant melanoma, metastatic melanoma, biomarker, serum/tissue biomarker in melanoma, melanoma therapy, melanoma immunotherapy, targeted therapy in melanoma, S100 melanoma, lactate dehydrogenase (LDH) melanoma, Braf melanoma, and diagnostic markers. All articles used in this review were peer reviewed. Markers only reported in a single article (with the exception of the immune inflammatory IL-8 and IgG4) were excluded, and markers reported in the literature only before the year 2000 were excluded.

**Table 2:** **Selected biomarker studies in melanoma**.

Biomarker	Study cohort	Correlation	Methodology^a^	Reference
LDH	50 patients stage I–II and 61 patients stage IV before and after treatment	Tumor stage; prognosis	Photometric assay	Egberts (41)
	30946 patients stage I–III and 7972 stage IV	Survival rate	Meta-analysis	Balch (4)

Tyrosine	200 patients stage IV	Prognosis	Nested RT-PCR^b^	Quaglino (42)
	114 patients stage I–IV and 20 healthy volunteers	Survival rate	RT-PCR	Visus (43)
	201 patients stage I–IV and 40 healthy volunteers	Overall survival	RT-PCR	Samija (44)

COX-2	63 human melanocytic skin tumors (17 nevi; 36 primary cutaneous melanomas; 11 lymph-node metastasis)	Tumor progression	IHC^c^	Kuzbicki (45)
	101 primary melanomas and 28 metastatic	Breslow thickness	IHC	Becker (46)

cMMP-1 and MMP-3	70 melanomas	Disease-free survival	IHC	Nikkola (47)

MMP-9	71 patients stage IV and 8 healthy volunteers	Poor prognosis	ELISA^d^	Nikkola (48)

MMP-2	482 melanoma (330 primary and 152 metastatic); 149 nevi (49 normal and 100 dysplastic)	Tumor progression	IHC	Rotte (49)

VEGF	125 blood samples from patients and 30 healthy volunteers	Tumor stage; survival	ELISA	Ugurel (50)
	155 melanoma blood samples	Tumor progression	RT-PCR	Quaglino (42)
	324 melanoma blood samples	Tumor stage	ELISA	Pelletier (51)

VEGF-C and VEGFR-3	75 melanoma blood samples (stage IV) and 30 healthy volunteers	Tumor burden	ELISA	Mouawad (52)

Osteopontin	345 melanoma patient blood samples	Breslow thickness and survival	IHC	Rangel (53)
	34 invasive growing melanomas	Poor prognosis	IHC	Alonso (54)
	106 patient blood samples	Tumor stage	ELISA	Maier (55)

Gal-3	53 benign nevi; 31 dysplastic nevi; 59 *in situ* melanoma; 314 primary melanoma; 69 metastatic melanoma	Tumor progression	IHC	Brown (56)

YKL-40	110 melanoma patient blood samples (stage IV) and 245 healthy volunteers	Tumor progression	ELISA	Schmidt (57)
	234 melanoma blood patient samples (stage I–II)	Poor prognosis	ELISA	Schmidt (58)
	50 melanoma patient blood (stage I–II) and 61 (stage IV)	Tumor stage	ELISA	Egberts (41)

MIA	110 with advanced disease and 66 disease free and 65 healthy controls	Survival rate	ELISA	Diaz-Lagares (59)
	125 melanoma patient blood sample	Poor prognosis	ELISA	Essler (60)

CEACAM	49 melanoma patient blood (stage III–IV)	Tumor stage; survival	ELISA	Sivan (61)

S100B	221 melanoma patient blood (stage II–III)	Survival rate	Line Immunoassay	Bouwhuis (62)
	20 melanoma patient blood	Metastasis detection	ELISA	Oberholzer (63)
	192 melanoma patient blood	Poor prognosis	ELISA	Beyeler (64)

IgG4	33 melanoma patients	Progression, overall survival	Luminex	Karagiannis (65)

IL-8	16 melanoma patients	Tumor burden, stage; survival	ELISA	Sanmamed (66)

*^a^Applies to laboratory assay unless the reference was a meta-analysis*.

*^b^Reverse-Transcription Polymerase Chain Reaction*.

*^c^Immunohistochemistry*.

*^d^Enzyme-Linked Immunosorbance Assay*.

### Serum biomarkers for melanoma

#### Lactate dehydrogenase

The enzyme LDH is a clinical serological biomarker in melanoma. It remains to date the strongest prognostic indicator found to be elevated with tumor burden (67). LDH is mostly released upon cell damage or death, with both phenomena indicating higher tumor burden and disease progression. An increase in serum LDH is, however, not specific to malignancy, but also occurs in many other settings such as hemolysis, infection, infarction, and inflammation. Therefore, the positive predictive value in melanoma is limited by this false-positive rate (68). Recent studies have shown that LDH is less sensitive in early stage disease, but has negative predictive value for metastatic relapse (69–71). It is nonetheless at present a useful and clinically available tool for indication of tumor progression in patients at later disease stages and it is therefore incorporated in the TNM classification (Table 1).

#### Tyrosinase

Tyrosinase is part of the biosynthesis process of melanin and is constitutively expressed in melanocytes and melanoma cells. Tyrosinase mRNA levels are detectable in the blood of melanoma patients with advanced metastatic disease detected by nested RT-PCR (42, 43). Initial evaluations revealed that tyrosinase is an independent prognostic marker for tumor progression (42, 43, 72). Samija et al. demonstrated that tyrosine mRNA is associated with a decrease in OS (44). However, high variability in the levels of serum tyrosinase have been reported, most likely due to difficulties in sample processing and the transient presence of metastasizing tumor cells in the blood (43). Therefore, it is not surprising that several studies could not confirm a significant prognostic utility for tyrosinase (73). This includes a recent study that showed no differences in tyrosinase serum levels when comparing patients to healthy volunteers (74).

#### Vascular endothelial growth factor

Growth factors in combination with interleukins are major regulators of inflammatory conditions in the tumor microenvironment. Vascular endothelial growth factor (VEGF) is known to support tumor-associated angiogenesis, and to contribute to inflammatory conditions, which promote immunosuppression and redirection of effective anti-tumoral immunity. Ugurel et al. reported that VEGF was an independent prognostic marker for overall and progression-free survival in their cohort (125 stage I–IV patients) (50). Unfortunately, several subsequent studies could not confirm this finding. Although associations between VEGF and the disease were found, no correlation with disease progression was attained (51, 75).

#### Osteopontin

Osteopontin, a secreted integrin-binding glycol-phosphoprotein, has been described to reduce apoptosis, enhance tumor growth, and be a major component for the recruitment of tumor-promoting stromal cells from the bone marrow (76, 77). In a recent study, Maier et al. demonstrated that osteopontin in combination with S100B can help to differentiate patients who are likely to subsequently relapse and develop metastatic disease from those who do not (55). Yet, the presence of osteopontin is also associated with other medical conditions such as autoimmune diseases and this may translate to false-positive readouts in patients with melanoma.

#### YKL-40

YKL-40 is a glycoprotein secreted by many cells including cancer cells and by immune cells such as macrophages and activated neutrophils (78–80). The physiological functions of YKL-40 are yet not fully understood and reports of its functionality as an independent prognostic marker vary (41, 57–59). More importantly, immunomodulatory drugs such as IL-2 and IFN-α2b stimulate YKL-40 expression and therefore its use as a biomarker can yield false-negative readouts during treatment (81).

#### Melanoma-inhibitory activity protein

The small protein melanoma-inhibitory activity (MIA) is secreted by melanoma cells and is involved in cell–cell contact by interacting with the extracellular matrix. It is also thought that MIA promotes tumor cell invasion and metastasis (82). Equally, it was reported that MIA has a high sensitivity and specificity compared to other clinically relevant biomarkers such as LDH (59, 69). Elevated levels of MIA correlated with more advanced disease stages, poorer prognosis, and decreased DFS (59, 69, 77, 83). However, different studies using multivariate analysis demonstrated higher rates of false-positive readouts in women, suggesting an alternative source of MIA may exist and that this protein may participate in and be influenced by other biological processes unrelated to malignancy (60, 69, 84).

#### S100

The family of S100 proteins has been of special interest as diagnostic markers in melanoma over the last decade, since the expression and secretion of these proteins is much higher in malignant compared to healthy tissues (85–87). S100 proteins and in particular S100B were recently found to be elevated in the serum of melanoma patients with these elevated levels being associated with poorer prognosis (63, 64), DFS, and OS (88). In a more recent prognostic study of patients treated with IFN-α2b versus observation in stage II and III patients, investigators showed that S100B is associated with worse OS and distant metastasis-free survival (DMFS). Interestingly, S100B in patients’ sera correlated over time with disease progression, increasing further in parallel to increases in disease burden (62). False-positive results can occur after brain, liver, or renal injury as well as during infectious diseases (89–91). However, data acquired to date point to the merits of further evaluation of S100 as a potential clinical tool.

#### IL-8

Interleukin-8 (IL-8), also known as CXCL8, is a chemokine produced by malignant cells and associated with inflammatory responses; it can induce neutrophil chemotaxis and is also thought to be a potent promoter of angiogenesis. A recent study has shown that IL-8 correlated with tumor burden, stage, survival, and response to therapy such as BRAF inhibitors (66). These data show that IL-8, perhaps in combination with other chemokines and cytokines that participate in tumor inflammation, has potential as a prognostic marker and warrants further investigation.

### Tissue-specific biomarkers for melanoma

Tissue specific biomarkers are molecules demonstrated to be over-represented in cancer lesions, and may facilitate diagnosis, early detection, prognosis of disease progression, and patient stratification.

#### Cyclooxygenase-2

Cyclooxygenases (COX1–3) are a group of proteins that are important modulators in the human body, affecting essential pathways such as the catabolic metabolism. COX1–3 also converts arachidonic acid into prostaglandin. Of this group, COX-2 can be induced in tumor cells (92, 93). Becker et al. showed a correlation between COX-2 staining intensity and Breslow thickness in melanoma (46). Furthermore, Kuzbicki et al. reported a higher COX-2 staining intensity in melanoma lesions compared to benign nevi (45).

#### Galectin-3

The Galectin-3 molecule is mainly secreted by inflammatory cells and is associated with both tumor progression and metastasis in melanoma (94). However, using a multifactorial Cox regression analysis, Brown et al. showed an inverse association between galectin-3 with tumor size (thin tumors had more galectin-3) and improved OS (56).

#### Matrix metalloproteinases

Matrix metalloproteinases play an important role in remodeling the tumor tissue microenvironment as they are responsible for proteolytically breaking components of the local tissue architecture, promoting tissue remodeling, and facilitating tumor cell migration (95, 96). For these reasons, the proteins are over-represented in tumor tissues. Nikkola and colleagues showed that MMP-1- and MMP-3-positive melanoma metastases correlate with decreased DFS (47). Furthermore, Rotte et al. confirmed a higher expression of MMP-2 in melanoma when compared to normal and dysplastic nevi. In this study, the levels of MMP-2 expression positively correlated with tumor progression and worse survival (49). It is, however, noteworthy that this study was conducted using tumor tissue microarrays through the application of peroxidase developed with 3,3’-diaminobenzidine (DAB – a brown substrate), making the assessment of MMP-2 difficult to distinguish from melanoma cells in pigmented lesions.

#### Cell adhesion molecules

Cancer-associated cell adhesion proteins are proteins that adhere to different cells, show altered expression levels in cancer, and may participate in tumor cell migration, immune cell evasion, and angiogenesis (97). Molecules such as the carcinoembryonic antigen-related cell adhesion molecule 1 (CEACAM-1) are known to foster interactions between somatic cells and immune cells. It was demonstrated that expression of CEACAM on tumor cells inhibits immune responses and leads to tumor progression (61). Investigation of CEACAM-1 expression suggested that it may be an independent factor to predict the risk of metastasis (98). Studies have also suggested associations between serum levels of cell adhesion molecules with the development of metastasis (99–101).

#### The chondroitin sulfate proteoglycan 4 as a biomarker

The chondroitin sulfate proteoglycan 4 (CSPG4), also known as the high molecular weight melanoma-associated antigen (HMW-MAA) or the melanoma-associated chondroitin sulfate proteoglycan (MCSP) is a glycoprotein–proteoglycan complex expressed on the surface of melanoma, glioma, neuroblastoma, certain breast carcinomas, and acute leukemias (102). CSPG4 is thought to play crucial roles in cell adhesion, melanoma migration, and metastasis (103). Although CSPG4 is over-expressed in over 80% of all melanomas, it is found at all disease stages and data so far have not yielded any correlations with disease progression (104). However, a recent study reported that a cytoplasmic form of the protein appears to correspond with response to treatment with a melanoma vaccine, indicating that CSPG4 may potentially be evaluated as a marker of response or progression (105).

## The Immune Response as a Source of Biomarkers

Cancer development and progression is associated with dysregulated molecular pathways in tumor cells, but it is now accepted that it may also be affected by cross-talk with the immune response. The latter could lead either to immune cell activation and destruction of cancer cells or to immune response redirection and suppression to support cancer cell survival and metastasis (106). Different immune sentinels and molecules associated with tumor inflammation with either possible tumor-promoting or tumor-eradicating effects may be evaluated as potential biomarkers to predict clinical course or to monitor how patients respond to treatments (107). For example, the immune checkpoint molecule programed death-ligand 1 (PD-L1), which is also expressed in melanoma and other cancers has been described in preliminary studies to identify enhanced aggressive and invasive disease and to be an independent risk factor for worse clinical outcome (108). Furthermore, gene expression profiling analyses suggest that high expression of immunological parameters may predict responses to anti-CTLA-4 antibody therapies (109).

The potential value of immune sentinels in cancer could be exemplified by reports that monitoring tumor infiltrating lymphocytes (TIL) (immune score) may be more accurate prognostically than standard methods such as TNM stage (110, 111). Further analysis into the phenotype of the TIL revealed that while cytotoxic T cells may predict better patient outcomes and responses to checkpoint blockade therapies, high numbers of regulatory T cells (Treg: CD4^+^FoxP3^+^CD25^high^) among TIL, or the presence of myeloid-derived suppressor cells (MDSC) may correlate with poorer survival in many cancers and with lower clinical response rates to anti-CTLA-4 antibodies (109, 112–114). Although most clinical studies are focused on T cells, emerging evidence also points to a potential prognostic value for B cells in cancer. For instance, the presence of CD20+ cells among TIL correlated with a more favorable prognosis in ovarian cancer, while other studies point to immune regulatory roles for B cells (115). These findings suggest that the specific immune status of patients may be indicative of capacity to respond to and possibly restrict tumor progression.

### Immunoglobulins: Antibody class bias and tumor-reactive responses

Early disease stages are characterized by mostly minimal disease burden with no more than 1 × 10^6^ tumor cells, which translate to very low or undetectable serum levels of potential biomarkers. Unless actively secreted, molecules released from these few cells may not be easily detectable in the circulation, thus monitoring such markers could be challenging.

B cells can undergo class switching to produce affinity matured antibodies in response to tissue damage and to the presence of antigens, including cancer antigens, and this may happen at early stages of cancer development. An individual B cell can produce 5,000–20,000 antibodies/min, and B cells can go through mitosis every 3 days, which can maintain or further enhance antibody production (116–118). The potential specificity of B cell and antibody responses to tumor antigens and amplification of the “tumor signal” may fulfill all three desirable attributes of a biomarker, namely detectability, sensitivity, and specificity. In support of this notion, the immunoglobulin G kappa chain has been shown to be a positive prognostic biomarker in breast cancer, non-small lung cancer, and colorectal cancer, not only for monitoring disease progression but also for predicting the response to neoadjuvant chemotherapy (115, 119, 120).

Until recently, the nature and functional significance of the humoral response in cancer were poorly understood. Moreover, whether and how the humoral response may be “immunoedited” by tumors has remained unknown. We have reported a tumor-reactive mature memory B cell compartment in the circulation of patients with melanoma. By isolating tumor-reactive antibody clones from patients’ mature B cells, we found that patient-derived antibodies can activate effector cells to destroy tumor cells *ex vivo* by recognizing tumor antigens on the surface of these cells (121). These findings suggest that host humoral immunity recognizes the presence of cancer and may potentially be activated. Antibodies, whether administered as therapeutics or expressed by host B cells, have the potential to induce potent anti-tumoral responses by sequestering effector cells to destroy tumor cells. However, since it is known that immunoediting in the tumor microenvironment may suppress the anti-tumoral functions of T cells, it would be reasonable to hypothesize that tumor-induced inflammatory conditions triggering suppression of T cell functions can lead to immunoediting of humoral responses as well and may substantially impair the potency of B cell and antibody functions against cancer.

Hints that immune escape mechanisms may operate to suppress tumor-reactive humoral responses came from our findings that this tumor-reactive memory B cell compartment appears more prominent at earlier disease stages and is reduced with disease progression. It has also been demonstrated that both the broad and the tumor-reactive mature memory B cell compartments are reduced in patients with advanced disease (122).

In melanoma, early studies indicated dysregulation and polarization of IgG antibody subclasses in the serum of patients with melanoma, but the biological relevance and functional implications of these findings were unclear (123). More recently, the presence of infiltrating IgG4+ plasma cells in hepatic cholangiocarcinomas (124) has been reported, pointing to diverted immunoglobulin class/subclass distribution in cancer. In support of these findings, we demonstrated that in melanoma tumor microenvironments B cells are polarized to favor production of IgG4, an antibody subclass with substantially restricted effector functions compared to well-known potent IgG1 antibodies and with capacity to impair effector functions of tumor-reactive IgG1 antibodies (65). This bias may occur in “alternative” Th2 cytokine environments in melanoma, where the presence of the immunoregulatory cytokine IL-10 can favor class switching to IgG4 and can enhance IgG4 production by class-switched B cells in the presence of IL-4 (125, 126). This biological relevance of IgG4 subclass antibodies in disease pathogenesis adds to the relatively newly described roles of IgG4 subclass in some inflammatory diseases (IgG4-related disease) (127).

Tumor antigen recognition may be another attribute of antibodies that could render them promising potential biomarkers. This may be facilitated by emerging screening platforms such as “immunosignaturing,” which is designed to identify reactive antibody signatures against panels of antigens, including those found in cancers. The “signatures” resulting from this process could allow for the identification of antibody reactivity patterns that may predict disease course. The potential for this approach has been demonstrated in the diagnosis of Alzheimer’s disease (128) and to detect antibodies against brain tumor antigens (129), but it may also be evaluated as a clinical biomarker tool in other cancers including melanoma.

Different facets of antibodies, including tumor specificity and reactivity, as well as antibody class and subclass bias may therefore be examined in the context of cancer including; as measures of the potency of adaptive immunity in cancer, as readouts of tumor-induced immunoediting and as powerful therapeutic agents to activate patient immune cells against cancer (35). Host humoral responses associated with tumor-immune escape may be linked to a higher risk of disease relapse and could point to readily detectable biomarkers, possibly at early disease stages.

## Concluding Thoughts

Biomarkers that move beyond the current clinical pathological and radiological parameters, helping to identify those patients with early disease at high risk of relapse and guiding therapy choices for patients with metastatic disease, are still needed. A number of potential candidate biomarkers, including immunological markers warrant further evaluation in melanoma. Circulating or tumor-resident immune cells, including those associated with immunosuppressive forces in melanoma such as Treg MDSC, IgG4+ B cells, and also cytokines, chemokines, checkpoint molecules, and antibodies may point to yet unexplored biomarker signatures associated with particular clinical outcomes. Despite the considerable progress made in immune monitoring technologies, it has been challenging to draw accurate correlations between immunological parameters and clinical outcomes or patient responses to therapeutic agents. The reasons might include complex interactions between immune and tumor cells and the variable patient immune responses, making it difficult to account for all the interactions required for adequate prognostic readouts. Even when associations with melanoma are demonstrated, there is significant variability among patients, possibly reflecting the heterogeneity of individual tumors and of individual patient immune responses.

Other important challenges for biomarker discovery and validation remain. As most biomarkers are normal cellular or immunological proteins, they can be detected in both healthy and disease states, reducing their specificity. Additionally, many studies report relatively small sizes of patient samples, and this may indicate a limitation potentially preventing biomarker selection and routine use. Future directions may include larger cohorts assessed in multi-center studies and also assessing combinations of multiple putative markers to identify specific prognostic footprints. Finally, complex statistical analyses and comprehensive algorithms will be needed in order to integrate these multiple lower specificity biomarkers.

In conclusion, the potential of biomarkers to contribute toward better clinical monitoring and management will most likely be aided through further refinement of emerging design, statistical, bioinformatics, and analytical methods (130), as well as via our enhanced understanding of disease pathways and tumor-immune cell cross-talk.

## Conflict of Interest Statement

The authors declare that the research was conducted in the absence of any commercial or financial relationships that could be construed as a potential conflict of interest.
